# The effect of liver interference on mammary tumour induction by 7,12-dimethylbenz(a)anthracene in Sprague-Dawley rats.

**DOI:** 10.1038/bjc.1967.23

**Published:** 1967-03

**Authors:** I. R. Kernohan, M. S. Inglis, D. N. Wheatley


### THE EFFECT OF LIVER INTERFERENCE ON MAMMARY TUMOUR INDUCTION BY 7,12-DIMETHYLBENZ(a)ANTHRACENE IN SPRAGUE-DAWLEY RATS

I. R. KERNOHAN, MARGET S. INGLIS AND D. N. WHEATLEY*

*From the Department of Pathology, University Medical Buildings, Foresterhill, Aberdeen*

Received for publication October 14, 1966

7,12-DIMETHYLBENZ(a)ANTHRACENE (DMBA) given in a single dose (30 mg.). in oil by stomach tube or intravenously (5 mg.) in lipid emulsion induces mammary tumours in Sprague-Dawley rats within 2–3 months (Huggins, Briziarelli and Sutton, 1959; Huggins, Grand and Brillantes, 1961), and also causes severe adrenal necrosis within 2–3 days (Huggins and Morii, 1961). We have shown that DMBA itself is probably not responsible for adrenal necrosis (Wheatley, Kernohan and Currie, 1966; Wheatley, Hamilton, Currie, Boyland and Sims, 1966) but must first be metabolized by the liver. Boyland and Sims (1965) found that DMBA is metabolized by rat liver mainly by hydroxylation of the methyl groups; 7-hydroxymethyl-12-methylbenz(a)anthracene is probably the adrenocorticolytic metabolite (Boyland, Sims and Huggins, 1965; Wheatley, Hamilton, Currie, Boyland and Sims, 1966). Boyland *et al.* (1965) tested the carcinogenicity of DMBA and its hydroxymethyl derivatives in rats and mice. Briefly, the 7-hydroxymethyl derivative induced mammary tumours but with a longer mean induction time than DMBA whereas the 12-hydroxymethyl derivative was not carcinogenic in rats although it was in mice.

It is also possible that a metabolite of DMBA, and not DMBA itself, is responsible for tumour induction. We have therefore tested the ability of DMBA to produce tumours in rats with impaired liver function.

### MATERIALS AND METHODS

Sprague-Dawley female rats from an accredited stock were obtained from Oxford Laboratory Animal Colonies, Oxford, England. $CCl_4$ (AnalaR) was given to 60 rats at a dose of 0·3 ml. of a 50% solution in olive oil intraperitoneally. Forty-three rats were partially hepatectomized, two-thirds of the liver being removed by the method of Higgins and Anderson (1931). Forty rats were given olive oil intraperitoneally (0·3 ml.) as controls. Twenty-four hours later the rats received 5 mg. DMBA in 1·0 ml. 15% cottonseed oil emulsion via a lateral tail vein. At the time of DMBA injection rats were 50 days old and weighed about 150 g.

The severity of the combined treatments resulted in a high early mortality. The surviving rats were palpated twice a week from the fourth week after injection. Tumours were measured and charted as described previously (Stevens, Stevens and Currie, 1965). The experiment was terminated at 6 months and an assessment

* Requests for reprints to Dr. D. N. Wheatley.

was made of tumour types from their growth curves and histological appearances as described by Stevens *et al.*, 1965.

### RESULTS

Rats with impaired liver function at the time of DMBA administration did not differ from intact healthy control rats in the percentage of rats developing tumours or in the tumour induction period (Table I). The incidence of rats bearing

TABLE I.—*Mammary Tumours in Rats Given DMBA After Liver Interfrence*

| Group | Number of rats with tumours | Total tumours | Average number tumours/ tumour-bearing rat | Mean induction time (weeks) |
|---|---|---|---|---|
| Control | 19/25 | 39 | 2·05 | 15·3 |
| $CCl_4$ | 25/29 | 64 | 2·56 | 15·1 |
| Partial hepatectomy | 17/24 | 36 | 2·12 | 15·9 |

mammary tumours 6 months after DMBA treatment varied between 70 and 86 per cent. (Fig. 1). This is lower than reported by others (e.g. Huggins, Grand and Brillantes, 1961 ; Tanaka and Dao, 1965) but in a number of our rats " tumours "

FIG. 1.—Percentage of mammary tumour-bearing rats against time after treatment of Sprague-Dawley female rats with 5 mg. DMBA intravenously.
—— control rats
· — · $CCl_4$-treated rats
– – – partially hepatectomized rats were palpated during life which proved on histological examination to be simple cysts. Table I also records the average number of tumours developing per rat. Although the rats pretreated with carbon tetrachloride developed more tumours than rats in the other two groups, the difference was not significant.

TABLE II.—*Growth Characteristics of Mammary Tumours in Rats Given DMBA After Liver Interference*

| Group | Total number of tumours | Growing | Static | Regressing* | Unclassified (found at necropsy) |
|---|---|---|---|---|---|
| Control | 39 | 13 | 12 | 9 | 5 |
| $CCl_4$ | 64 | 26 | 24 | 9 | 5 |
| Partial hepatectomy | 36 | 14 | 5 | 11 | 6 |

* In *each* group, 3 " tumours " are included which showed complete regression. Whilst they were almost undoubtedly mammary tumours, histological confirmation was not obtained.

The growth characteristics of the tumours are summarized in Table II. A greater proportion of tumours were either growing or regressing in the partial hepatectomy group, but with the small numbers involved no significant difference from controls was found.

Histologically, tumours in the partially hepatectomized rats and in the $CCl_4$-treated rats were of similar types to those found in the control group.

### DISCUSSION

Impairment of liver function at the time of administration of DMBA does not appreciably alter the incidence of Sprague-Dawley rats developing mammary tumours. If DMBA is metabolized to a powerful carcinogenic derivative, control rats would be expected to produce more tumours than the experimental groups, but this is not so. Since the metabolism of DMBA is considerably affected by the treatments employed in this experiment, rats will presumably be exposed to unaltered DMBA for longer than controls. An increased yield of tumours might be expected if DMBA itself is the potent carcinogen ; a slightly higher yield was found in $CCl_4$-treated rats but in partially hepatectomized rats there was a slightly lower yield than controls ; however, these differences are not significant. This suggests that DMBA itself is the primary carcinogenic agent and not a metabolite. Furthermore, since rats capable of efficiently metabolizing DMBA (control group) yielded overall a similar number of tumours to rats which could not metabolize DMBA so quickly, carcinogenesis must be initiated very early after the injection of the polycyclic hydrocarbon. This is in agreement with the findings of Dao, Tanaka and Gawlak (1964) who showed that mammary glands transplanted to untreated rats as soon as 6 hours after injection of DMBA developed tumours. It is considered that a certain amount of DMBA is absorbed by the quiescent mammary tissue soon after injection, and that the metabolism of the bulk of the remaining DMBA by the liver does not significantly influence tumour production.

Tanaka and Dao (1965) investigated the effect of a very small dose of $CCl_4$ (insufficient to protect rats from adrenal necrosis following DMBA treatment) on mammary tumours after 20 mg. DMBA intragastrically. Apart from perhaps a longer induction time, they report no other effect.

In another related study Helfenstein and Young (1963) investigated the effect of metyrapone (2-methyl-1,2-*bis*-(3-pyridyl)-1-propanone). Whilst this drug protected adrenal glands from the adrenocorticolytic action of DMBA, little difference in mammary tumour production was found between metyrapone-treated and control rats.

It can be concluded that the carcinogenic nature and the adrenocorticolytic action of DMBA are essentially unrelated phenomena. These experiments suggest that DMBA is the potent carcinogen and not a metabolite.

SUMMARY

Liver function was impaired in Sprague-Dawley female rats by carbon tetrachloride treatment or by partial hepatectomy 24 hours before treatment with 5 mg. 7,12-dimethylbenz(a)anthracene intravenously. Although these treatments are known to protect the adrenal glands by preventing the metabolism of DMBA to an active adrenocorticolytic derivative, they did not affect the incidence of rats developing mammary tumours or the relative frequencies of the tumours of different growth characteristics to any significant extent.

It is concluded that DMBA itself, and not a derivative, is probably responsible for mammary tumour induction.

This work was supported by the Scottish Hospital Endowments Research Trust Fund. We thank Professor Currie for his interest in this work and for his criticisms of the manuscript. DMBA in 15% lipid emulsion was prepared by Dr. Paul Schurr (The Upjohn Co., Kalamazoo, Michigan, U.S.A.) and was kindly provided by Professor Charles Huggins.

REFERENCES

Boyland, E. and Sims, P.—(1965) *Biochem. J.*, **95**, 780.
Boyland, E., Sims, P. and Huggins, C.—(1965) *Nature, Lond.*, **207**, 816.
Dao, T. L., Tanaka, Y. and Gawlak. D.—(1964) *J. natn. Cancer Inst.*, **32**, 1259.
Helfenstein, J. E. and Young, S.—(1963) *Nature, Lond.*, **200**, 1113.
Higgins, G. M. and Anderson, R. M.—(1931) *Archs Path.*, **12**, 186.
Huggins, C., Briziarelli, G. and Sutton, H.—(1959) *J. exp. Med.*, **109**, 25.
Huggins, C., Grand, L. C. and Brillantes, F. P.—(1961) *Nature, Lond.*, **189**, 204.
Huggins, C. and Morii, S.—(1961) *J. exp. Med.*, **114**, 741.
Stevens, L., Stevens, E. and Currie, A. R.—(1965) *J. Path. Bact.*, **89**, 581.
Tanaka, Y. and Dao, T. L.—(1965) *J. natn. Cancer Inst.*, **35**, 631.
Wheatley, D. N., Hamilton, A. G., Currie, A. R., Boyland, E. and Sims, P.—(1966) *Nature, Lond.*, **211**, 1311.
Wheatley, D. N., Kernohan, I. R. and Currie, A. R.—(1966) *Nature, Lond.*, **211**, 387.


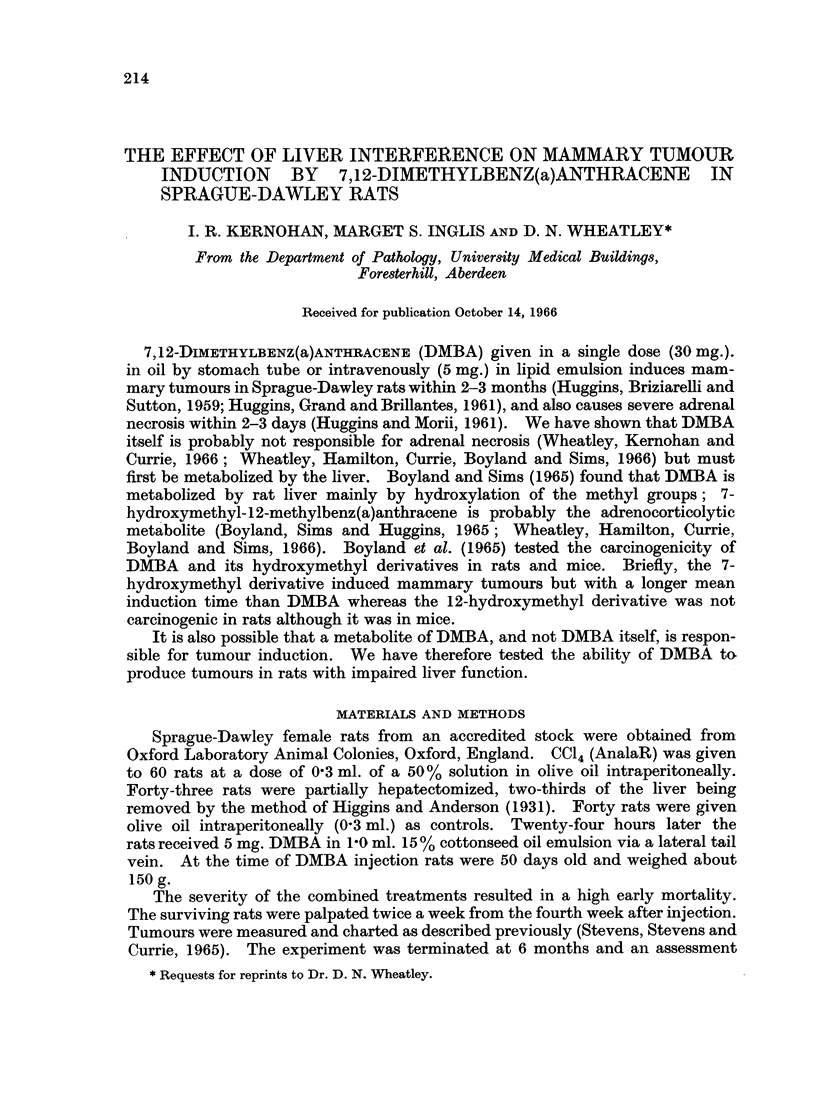

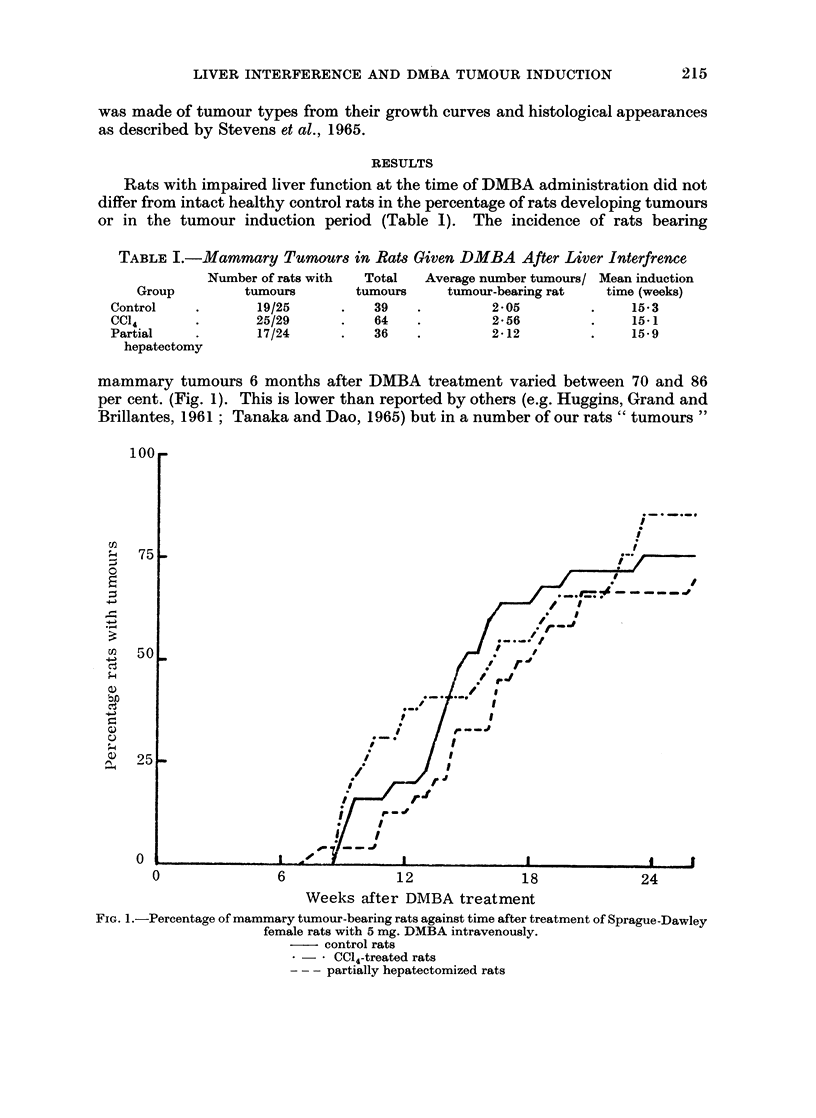

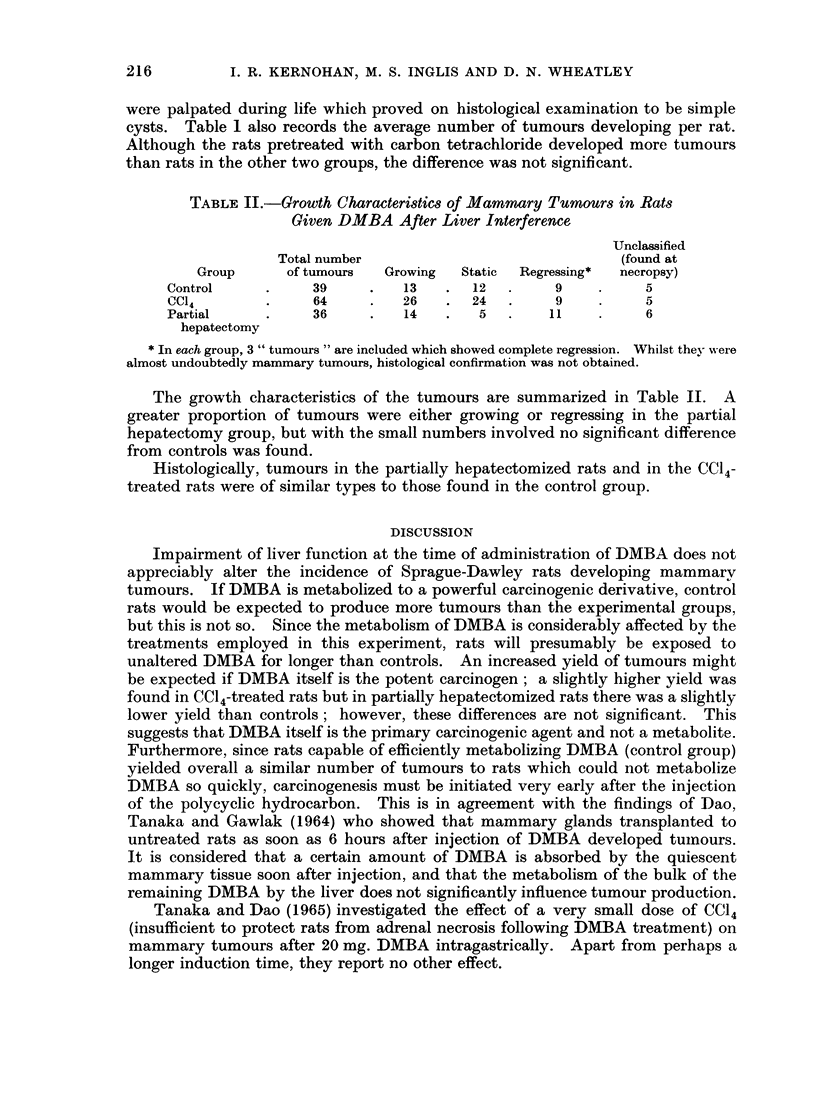

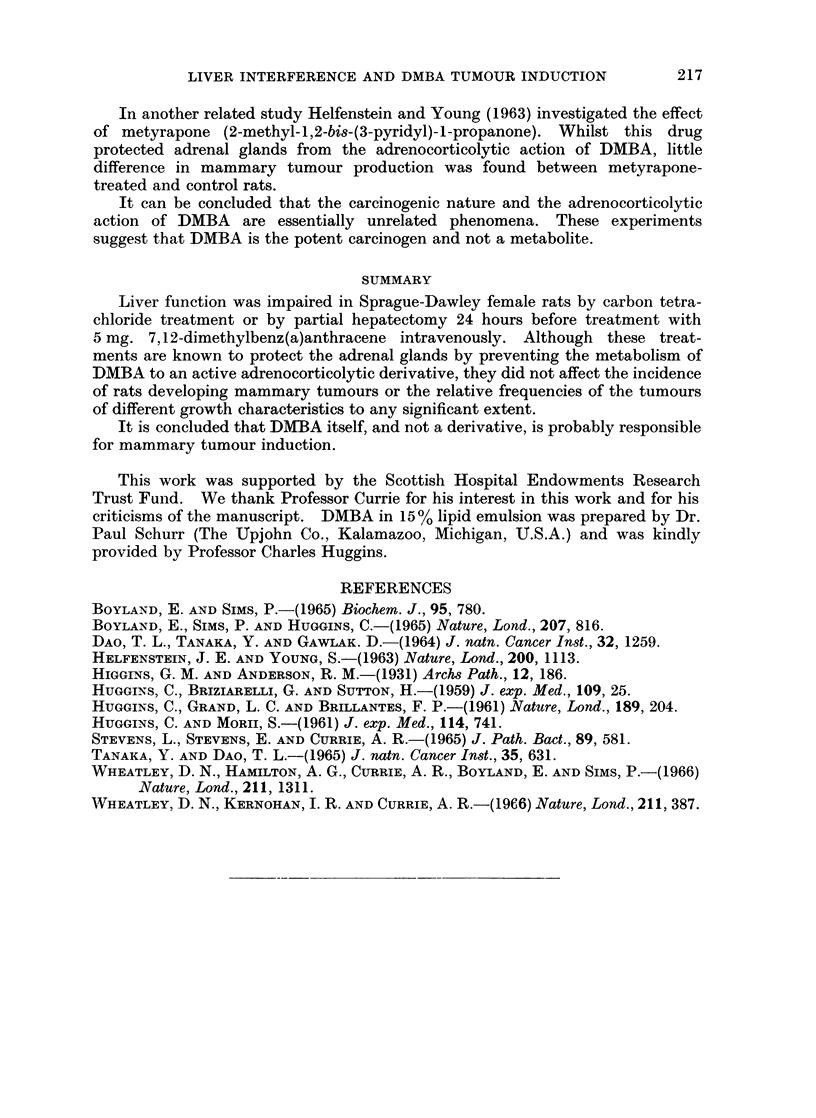


## References

[OCR_00221] Boyland E., Sims P., Huggins C. (1965). Induction of adrenal damage and cancer with metabolites of 7,12-dimethylbenz(a)anthracene.. Nature.

[OCR_00225] DAO T. L., TANAKA Y., GAWLAK D. (1964). TUMOR INDUCTION IN TRANSPLANTED MAMMARY GLANDS IN RATS.. J Natl Cancer Inst.

[OCR_00227] HELFENSTEIN J. E., YOUNG S. (1963). EFFECT OF 2-METHYL-1,2-BIS-(3-PYRIDYL)-1-PROPANONE (METYRAPONE) ON THE PRODUCTION OF MAMMARY TUMOURS INDUCED IN RATS BY ORAL FEEDING WITH DIMETHYLBENZANTHRACENE.. Nature.

[OCR_00231] HUGGINS C., GRAND L. C., BRILLANTES F. P. (1961). Mammary cancer induced by a single feeding of polymucular hydrocarbons, and its suppression.. Nature.

[OCR_00232] HUGGINS C., MORII S. (1961). Selective adrenal necrosis and apoplexy induced by 7, 12-dimethylbenz(a)anthracene.. J Exp Med.

[OCR_00234] STEVENS L., STEVENS E., CURRIE A. R. (1965). HISTOLOGICAL STUDIES AND MEASUREMENT OF NUCLEIC ACID SYNTHESIS IN RAT MAMMARY TUMOURS INDUCED BY 9,10-DIMETHYL-1,2-BENZATHRACENE (DMBA).. J Pathol Bacteriol.

[OCR_00235] Tanaka Y., Dao T. L. (1965). Effect of hepatic injury on induction of adrenal necrosis and mammary cancer by 7,12-dimethylbenz [alpha] anthracene in rats.. J Natl Cancer Inst.

[OCR_00237] Wheatley D. N., Hamilton A. G., Currie A. R., Boyland E., Sims P. (1966). Adrenal necrosis induced by 7-hydroxymethyl-12-methylbenz(a)anthracene and its prevention.. Nature.

[OCR_00241] Wheatley D. N., Kernohan I. R., Currie A. R. (1966). Liver injury and the prevention of massive adrenal necrosis from 9, 10-dimethyl-1,2-benzanthracene in rats.. Nature.

